# The multi-vessel and diffuse coronary spasm is a risk factor for persistent angina in patients received anti-angina medication

**DOI:** 10.1097/MD.0000000000013288

**Published:** 2018-11-21

**Authors:** Sang-Ho Park, Byoung Geol Choi, Seung-Woon Rha, Tae Soo Kang

**Affiliations:** a Cardiology Department, Soonchunhyang University Cheonan Hospital, Cheonan; b Department of Medicine, Korea University Graduate School, Seoul; c Cardiovascular Center, Korea University Guro Hospital, Seoul; d Cardiovascular Division, Department of Internal Medicine, Dankook University Hospital, Cheonan, Korea.

**Keywords:** acetylcholine provocation test, clinical outcomes, coronary artery spasm, type

## Abstract

Coronary artery spasm (CAS) is known to be a risk factor for cardiovascular events. However, there is limited data whether the multi-vessel and diffuse spasm (MVDS) is related to more adverse clinical outcomes compared to the Non-MVDS. The aim of this study is to evaluate the impact of the MVDS on clinical outcomes during a 3-year clinical follow-up period.

A total 2797 patients underwent coronary angiography (CAG) with acetylcholine (ACH) provocation test from Nov 2004 to Oct 2010 were enrolled. It is a single-center, observational, prospective, all-comers registry designed to reflect the “real world” practic. The patients were divided into the 3 groups; the negative spasm (NS) group (n = 1188), the Non-MVDS group (n = 1081), and the MVDS group (n = 528). The incidence of major adverse cardiac events (MACE) and recurrent angina was evaluated up to 3 years. To minimize confounding factors, multivariable Cox-proportional hazards regression analysis was performed.

In the 3-year clinical follow-up, the incidence of total death, myocardial infarction, de novo percutaneous coronary intervention (PCI), cerebrovascular accident and MACE were similar among the 3 groups. However, recurrent angina occurred more frequently in the MVDS group than in the NS group (hazard ratio [HR], 1.96; 95% confidence interval [CI], 1.27–3.02; *P* = .002). Recurrence angina between the MVDS group and the Non-MVDS group was not statistically significant (HR, 1.36; 95% CI, 0.91–2.03; *P* = .129).

In this study, although the incidence of major adverse cardiovascular events were not different regardless of spasm type, the MVDS was associated with higher incidence of recurrent chest pain requiring repeat CAG during the 3-year follow-up period, suggesting more intensive optimal medical therapy with close clinical follow up would be necessary for this particular subset of patients.

## 1 Introduction

Coronary artery spasm (CAS) without organic coronary stenosis is generally believed to have a good prognosis,^[1–4]^ it is often the major cause of chest pain which is closely related to ischemic heart diseases such as variant angina, unstable angina and myocardial infarction (MI).^[4–7]^ Recently studies, 33% to 79% of patients with chest pain without coronary artery disease (CAD) are diagnosed with CAS by the acetylcholine (ACH) provocation test.^[2,8]^ The Coronary Artery Spasm in Patients with Acute Coronary Syndrome (CASPAR) study is reported that 28% of the acute coronary syndrome (ACS) patients had no culprit lesion, and 49% of the patients were diagnosed with CAS by ACH test.^[5,6]^ Therefore, ischemic heart diseases due to CAS not only reduce the quality of life but also cause sudden death. Further, individual or combinations of vasomotion type such as focal or diffuse, single or multi-vessel spasm could show different clinical outcomes.^[3,9,10]^ Ong et al described the clinical features of vasomotion type, and Lee et al reported the impact of vasomotion type on long-term clinical outcomes.^[3,8]^ These studies showed that the epicardial CAS is not associated with hard cardiovascular events such as death, MI, and revascularization, but is associated with persistent chest pain during the follow-up period.^[3]^ Also, Onaka et al reported that a multi-vessel spasm is associated with a high risk of life-threatening cardiac events.^[9]^ Thus, the multi-vessel and diffuse spasm (MVDS) would be cause severe ischemic damage. However, the long-term clinical prognosis of MVDS has not been reported. In the present study, we investigated the impact of MVDS on long-term clinical outcomes in CAS patients who received optimal medical treatment for CAS.

## 2 Methods

The design of this registry has been introduced before.^[2,11–14]^ In brief, it is a single-center, observational, prospective, all-comers registry designed to reflect the “real world” practice since 2004. Data were collected by a trained study-coordinator with a standardized case report form. The participants or their legal guardians were given a thorough literal and verbal explanation of the study procedures before granting a written consent to participate in the study. Institutional Review Board (IRB) of Korea University Guro Hospital (KUGH) approved all of the consenting procedures. The authors of this manuscript have certified that the information contained herein is true and correct as reflected in the records of the IRB (#KUGH10045). This study has been specifically approved by the IRB of KUGH.

### 2.1 Study population

A total of 5882 consecutive patients presenting with typical or atypical chest pain who underwent coronary angiography (CAG) with ACH test from November 2004 to October 2010 at the Cardiovascular Center of KUGH, Seoul, South Korea were enrolled for this study. Among these, 2797 patients without significant CAD (defined as having a stenosis diameter of less than 70% on the quantitative CAG) underwent the ACH test. Patients were excluded if they had any of the following conditions; coronary artery bypass graft, prior percutaneous coronary intervention (PCI), prior cerebrovascular disease, advanced heart failure (New York Heart Association class III or IV) or serum creatinine ≥2 mg/dL, because these conditions could be major causes of adverse cardiovascular events and could bias the results.

### 2.2 Study definition

Significant CAS was defined as having greater than 70% luminal narrowing of the artery during ACH test regardless of ischemic EKG changes or presence of chest pain. The multi-vessel spasm was defined as having significant CAS in more than 2 major epicardial arteries. The diffuse spasm was defined as having a significant CAS with a site length of more than 20 mm. MVDS was defined as diffuse type spasm was developed in both left anterior descending and left circumflex coronary arteries during ACH test at any dose. The Non-MVDS was defined as any type of spasm of the coronary arteries following intracoronary ACH injection except for MVDS. The spontaneous spasm was defined as having focal or diffuse narrowing of greater than 30% in baseline CAG, compared to the reference vessel diameter after nitroglycerin administration into the intracoronary route. Deaths were regarded to be of cardiac cause unless a non-cardiac cause could be confirmed. Repeated CAG (mostly due to recurrent angina) was performed in patients who complained of recurrent angina despite adequate antianginal medication for at least 6 months since the onset of first CAG. Usually, repeat CAG was performed in patients with typical chest pain such as good response with nitroglycerin, effort-related chest pain and ambiguous recurrent ischemic symptoms to confirm the presence of recurrent significant CAS, mixed angina or newly developing advanced atherosclerotic CAD. Major adverse cerebrocardiovascular events (MACCE) were defined as the composite of total death, MI, stroke, or revascularization including PCI and coronary artery bypass graft.

### 2.3 ACH provocation test

The design of the ACH provocation test has been introduced before.^[2,11–14]^ Initial investigation for CAG included clinical history taking and non-invasive stress tests such as treadmill test, stress echocardiography, and radionuclide study. CAG was performed to confirm the presence of significant CAD. However, CAG was immediately done without functional studies in case of typical resting ischemic chest pain to confirm VSA. Vasodilators or vasoconstrictors such as nitrates, calcium channel blockers, beta blockers, nicorandil, and molsidomine were discontinued for at least 72 hours before CAG. CAS induction was tested by an intracoronary injection of ACH immediately after diagnostic angiography by either a trans-radial or trans-femoral approach. ACH was injected by incremental doses of 20 (A1), 50 (A2), and 100 (A3) μg/min into the left coronary artery over a 1-minute period with 5-minute intervals up to the maximally tolerated dose under a continuous monitoring with electrocardiogram and measuring of blood pressure. Routine provocation test of the right coronary artery was not done due to safety issues regarding the higher prevalence of advanced atrioventricular block, which needs a temporary pacemaker for maintaining adequate ACH infusion rate and cost-effectiveness for diagnosis and management of significant CAS. Angiography was repeated after each ACH dose until a significant focal or diffuse narrowing of greater than 70% was observed. If significant focal or diffuse vasoconstriction (>70%) of coronary arteries was induced at any dose, ACH infusion was stopped. An intracoronary injection of 0.2 mg of nitroglycerine was administered after completing the ACH provocation test, followed by CAG 2 minutes later. End-systolic images for each segment of the left coronary artery were chosen according to the corresponding points on the electrocardiographic trace (QRS onset or end of T wave) and analyzed using the proper QCA system of the catheterization laboratory (FD-20, Phillips, Amsterdam, The Netherlands). Coronary artery diameters were measured by QCA before and after the administration of ACH at the site that showed the greatest changes following drug administration. Reference vessel diameters were measured at the proximal and distal portions of each artery. The mean reference vessel diameter was used to assess diameter narrowing by QCA.

### 2.4 Statistical analysis

For continuous variables, differences between the 3 groups were evaluated by analysis of variance (ANOVA) or Kruskal–Wallis, and Post-hoc analysis between the 2 groups was evaluated by Hochberg or Dunnett-T3 test. Data were expressed as a mean ± standard deviation. For discrete variables, differences among the 3 groups were expressed as counts and percentages and analyzed with χ^2^ or Fisher exact test as appropriate. Multivariable Cox-proportional hazards regression analysis, which includes baseline confounding factors, was used for assessing independent impact factors. We tested all available variables that could be of potential relevance: age, sex, cardiovascular risk factors (hypertension, diabetes, dyslipidemia, current smokers, current alcoholics, and insignificant coronary stenosis), and myocardial bridge. Clinical outcomes were estimated with the Kaplan–Meier curved analysis and differences among the groups were compared with the log-rank test. A 2-tailed *P* value of <.05 was considered to be statistically significant. All statistical analyses were performed using SPSS 20 (SPSS Inc., Chicago, IL).

### 2.5 Study endpoints

The primary endpoint was the total death, MI, stroke, PCI, or MACCE. The secondary endpoint was the incidence of recurrent angina requiring repeat CAG, which is evaluated for 3 years. In this study, the mean follow-up period was 1095 days and we could follow up on the clinical data of all enrolled patients through medical chart reviews, telephone contacts and face-to-face interviews at the regular outpatient clinic.

## 3 Results

In the present study, a total of 2797 eligible patients without significant stenosis who underwent ACH test were enrolled in final, and of those 1609 patients were diagnosed with CAS. A total of 2797 eligible patients were divided into 3 groups: the negative spasm (NS) group (n = 1188), the Non-MVDS group (n = 1081), and the MVDS group (n = 528). The Non-MVDS group consisted of the single-vessel and focal-spasm (n = 238), the multi-vessel and focal spasm (n = 27), and the single-vessel and diffuse-spasm (n = 816).

**Figure 1:**
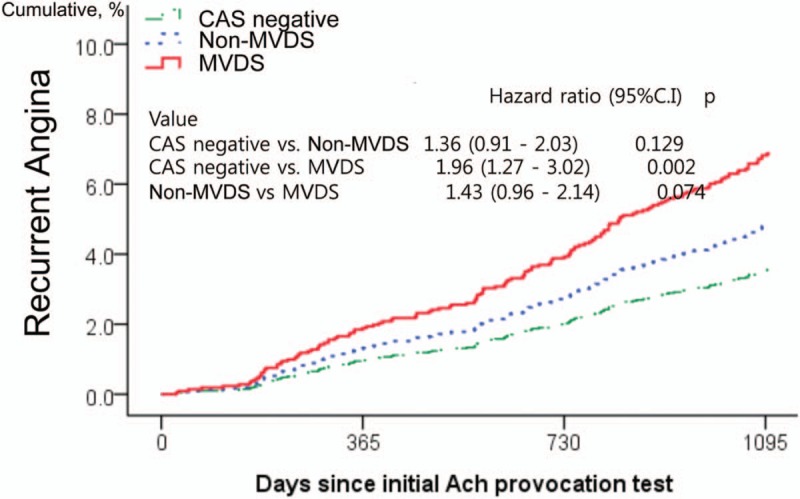
Survival curves analysis describing cumulative incidences of recurrent angina at 3 years. The figure shows a comparison of the incidence of recurrent angina between spasm types. In proportional hazard Cox-regression model analysis adjusted for covariates such as age, gender, hypertension, diabetes, dyslipidemia, current smoker, current alcoholics, myocardial bridge, and insignificant coronary stenosis, the multi-vessel diffuse spasm group (MVDS, red) had significantly higher recurrent angina risk [hazard ratio; 1.96, 95% confidence interval; 1.27 to 3.02, *P* = .02] than the CAS negative group (green) during 3-year follow-up period. However, Non-MVDS group (blue) did not show the significant difference in recurrent compared to negative CAS (HR, 1.36; 95% CI, 0.91–2.03; *P* = .129). MVDS group had a trend toward higher incidence of recurrent chest pain, compared to Non-MVDS group (HR, 1.43; 95% CI, 0.96–2.14; *P* = .074). ACH = acetylcholine, CAS = coronary artery spasm, MVDS = multi-vessel diffuse spasm.

The baseline characteristics and laboratory findings among the 3 groups as an enrolled patient are shown in Table 1. The baseline characteristics showed considerable differences among the 3 groups. Compare with the NS group, both of the MVDS group and Non-NVDS group had more an old age, males, alcohol drinkers, and smokers, and had a lower level of HDL-cholesterol. Also, the clinical and angiographic characteristics during the ACH test are shown in Table 1. Compare with the NS group, both the MVDS and the Non-MVDS groups were more had an insignificant coronary stenosis (<50%), and frequency of spontaneous spasm, myocardial bridge and ischemic ECG change such as an ST-segment depression, ST-segment elevation, and T-inversion. Especially, spontaneous spasm and ischemic EKG changes occurred more frequently in the MVDS group than in the Non-MVDS group. However, the myocardial bridge had more in the Non-MVDS group than in the MVDS group. The cumulative clinical follow-up result for 3-year are shown in Table 2. The primary endpoint, total death, MI, PCI, stroke, and MACCE were not significantly different among the 3 groups. However, a secondary endpoint, recurrent angina, is occurred higher in both the MVDS group and the Non-NVDS group as compare than the NS group. In Cox-proportional hazards regression model analysis, the MVDS group had significantly higher recurrent angina risk [hazard ratio (HR); 1.96, 95% confidence interval (CI); 1.27–3.02, *P* = .02] than the NS group, [Fig. 1] The Predictors for recurrent angina in the 3-year follow-up results are shown in Table 3. Independent predictors of recurrent angina were MVDS, aging, dyslipidemia, drinking habit and insignificant coronary stenosis less than 50% in Cox-proportional hazards regression model analysis.

**Table 1:**
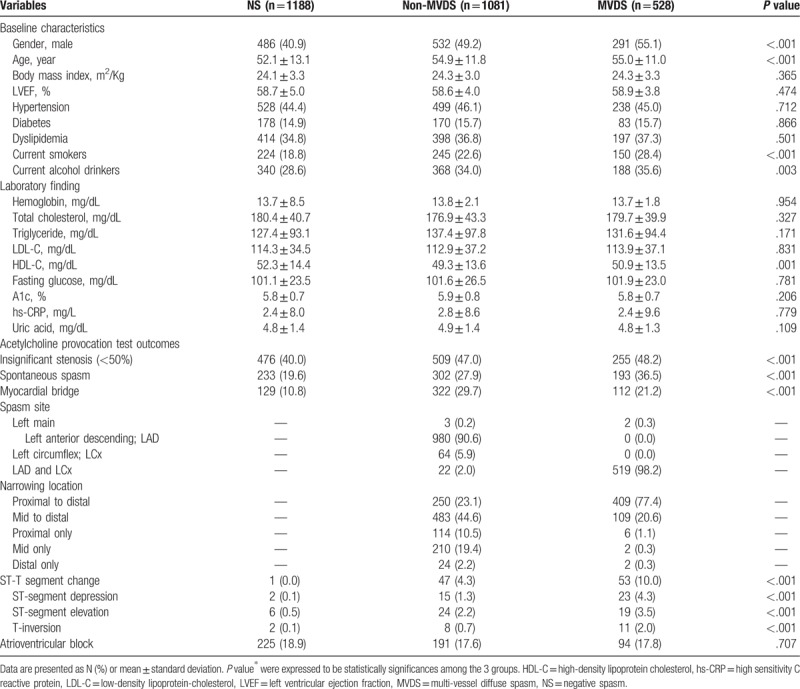
Baseline characteristics and angiographic parameters during the acetylcholine provocation test.

**Table 2:**
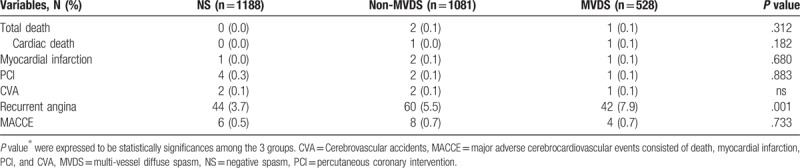
Cumulative clinical outcomes up to 3-year.

**Table 3:**
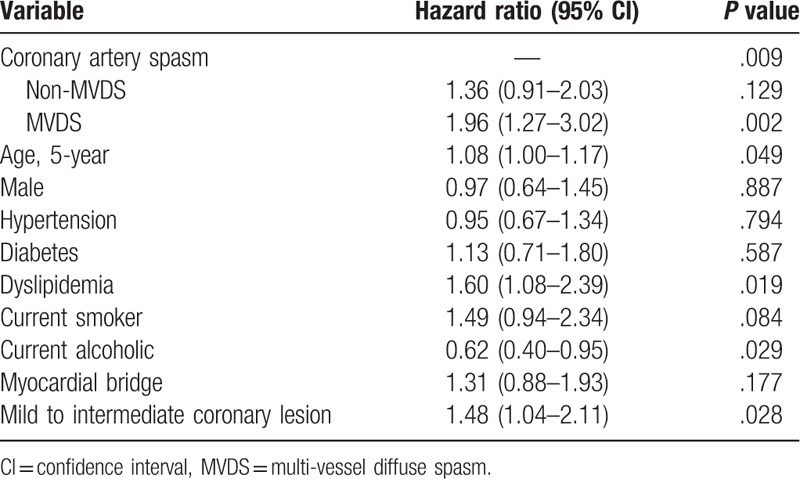
Predictors of recurrent angina requiring repeat coronary angiography.

## 4 Discussion

The narrowed coronary arteries due to CAS and CAD would cause ischemic damage to the myocardium, which can cause persistent chest pain, ACS, MI, and sudden death.^[1,5,7,9,13]^ It is well-documented that coronary stenosis is a predictor of the major adverse cardiac event. In particular, the multi-vessel disease has a poorer clinical prognosis than the single-vessel disease.^[15–18]^ Also, coronary stenosis >70% or diffuse-long lesion are well known as severe coronary stenosis.^[19]^ However, the clinical evaluation of the type of epicardial CAS is very limited. CAS is a well-known endothelial dysfunction, which can cause ischemic conditions, even if not a fixed organic lesion, by limiting the coronary flow.^[19–22]^ Therefore, CAS would be a major cause of angina and ACS. Especially, epicardial CAS is known to cause frequent angina episodes even with long-term anti-anginal medications.^[1,2,6]^ Thus, Clinical impact assessment according to the classification of epicardial CAS is needed.

In this study, we hypothesized that an epicardial MVDS would have the greatest effect on the clinical symptoms of angina when long-term clinical results among three groups of NS, MVDS, and Non-MVDS were followed up. Park et al reported that multi-vessel spasm was associated with clinical symptoms such as spontaneous spasm, the frequency of diffuse or severe spasms, chest pain and ischemic ECG changes during ACH test.^[10]^

In the present study, significant CAS occurred at 57.5% (1609/2797), of which 34.5% were a multi-vessel spasm, 83.5% were diffuse spasm and 32.8% MVDS by the ACH test in patients with suspected vasospastic angina. As in previous studies, the CAS such as MVDS or Non-MVDS had a more frequent recurrence of angina but was not associated with the increase of the hard clinical outcomes such as death, MI, de novo PCI, stroke, and MACCE during 3-year clinical follow-up, compared to the Non-CAS.^[2,6]^ In this study, CAS was subdivided into MVDS and Non-MVDS. The MVDS group had significantly higher recurrent angina risk (HR; 1.96, 95% CI; 1.27–3.02) than the NS group, not the Non-MVDS. (Fig. 1) Consequently, MVDS was the potential risk factor for recurrent angina along with aging, dyslipidemia, current alcohol, and mild to intermediate coronary stenosis. (Table 3)

MVDS is maybe affected by sex, body mass index, age, hyperlipidemia, alcohol, and high sensitivity C-reactive protein levels.^[10,23–25]^ These variables overlap with the potential risk factor for recurrent angina in Table 3. According to the results of this study, the more optimal medical therapy and closer clinical follow-up could be required in the epicardial CAS such as MVDS to achieve better prognosis. Also, in patients with MVDS, modification in lifestyle such as abstinence from alcohol, smoking, and statin use or changes in eating habits to control dyslipidemia could be an important factor to manage angina.^[12,20]^ In addition, the choice of medicines with anti-anginal effects in the treatment of co-morbidity disease such as hypertension or dyslipidemia for CAS patients may be considered to improve angina symptoms.^[11,26]^

In this study, there were several limitations. First, the present study is a non-randomized trial analysis conducted at a single center, and it is analyzed retrospectively and multivariable Cox-proportional regression analysis was performed to minimize the confounding factors which might have influenced the results. Also, the registry was designed with an all-comers prospective registry from 2004. We could not adjust all the limiting factors not shown through medical records or collected through telephone contact. Second, the ACH test can identify epicardial CAS, but microvascular CAS confirmation should be symptomatic. In this study, only epicardial CAS was defined as CAS. Previous studies have defined CAS from 70% narrowing of the coronary artery to subtotal or total occlusion.^[2,5,6,8,9,11–13,27,28]^ The present study, significant CAS was defined as having greater than 70% luminal narrowing of the artery during ACH provocation test. Though 70% narrowing may be less stringent than total occlusion, the more the severe spasm can be the higher chance of hemodynamic instability and advanced atrioventricular block can be developed. Because the most of the ACH provocation test was performed at the outpatient base by the 4F radial approach, patient safety was regarded as the first priority. Third, Routine the myocardial stress test such as SPECT (single-photon emission computed tomography), exercise treadmill, and dobutamine stress echocardiography was not performed because of the assumption of very limited possibilities of significant fixed coronary artery stenosis in patients suffering from mainly resting ischemic chest pain suspicious of vasospastic angina, not effort-induced angina. Finally, we could not gather any detailed follow-up data on anti-angina medication during the follow-up. However, all patients received anti-angina medication until free of angina symptom and clinical remission, although medication type and duration were at an individual physician's discretion. Further well-designed and more long-term follow-up studies are needed to get the more accurate answer.

## 5 Conclusion

In this study, all CAS patients received anti-anginal medications until free of angina symptoms and clinical remission. All the vasospastic patients were strongly recommended to maintain lower doses of anti-anginal medications for safety during follow-up. The main findings of the present study were; Regardless of the vasomotion type, CAS did not increase in the primary endpoint such as death, MI, de novo PCI, stroke, and MACCE. However, secondary endpoint, recurrent angina, more occurred in CAS patients, and MVDS was a most strong predictor of recurrent angina in a 3-year follow-up. Independent predictors of recurrent angina were MVDS, aging, dyslipidemia, drinking habit, and insignificant coronary stenosis less than 50%. Therefore, it would be safe and effective with optimal medical therapy and close clinical follow up for the patients with MVDS to achieve better clinical course.

## Author contributions

S-WR conceived and designed the research; S-HP, BGC, drafted the manuscript; S-HP, BGC, performed statistical analysis; TSK made critical revision of the manuscript for key intellectual content.

**Conceptualization:** Byoung Geol Choi, Sang-Ho Park, Seung-Woon Rha, Tae Soo Kang.

**Data curation:** Byoung Geol Choi.

**Formal analysis:** Byoung Geol Choi.

**Investigation:** Byoung Geol Choi, Seung-Woon Rha.

**Methodology:** Byoung Geol Choi.

**Project administration:** Sang-Ho Park, Seung-Woon Rha.

**Software:** Byoung Geol Choi.

**Supervision:** Seung-Woon Rha, Tae Soo Kang.

**Validation:** Byoung Geol Choi.

**Writing – original draft:** Sang-Ho Park.

**Writing – review & editing:** Byoung Geol Choi, Seung-Woon Rha, Tae Soo Kang.
